# Periapical status in patients affected by osteoporosis: A retrospective clinical study

**DOI:** 10.1002/cre2.604

**Published:** 2022-06-14

**Authors:** Erika Cadoni, Francesca Ideo, Giuseppe Marongiu, Silvia Mezzena, Luca Frigau, Quirico Mela, Antonio Capone, Henry F. Duncan, Elisabetta Cotti

**Affiliations:** ^1^ Department of Conservative Dentistry and Endodontics, University of Cagliari Cittadella Universitaria di Monserrato Monserrato Cagliari Italy; ^2^ Department of Surgical Sciences, University of Cagliari Cittadella Universitaria di Monserrato Monserrato Cagliari Italy; ^3^ Department of Economics and Business Sciences University of Cagliari Cagliari Italy; ^4^ Department of Medical Sciences and Public Health, University of Cagliari Cittadella Universitaria di Monserrato Monserrato Cagliari Italy; ^5^ Division of Restorative Dentistry and Periodontology, Dublin Dental University Hospital Trinity College Dublin Dublin Ireland

**Keywords:** apical periodontitis, bisphosphonates, denosumab, osteoporosis

## Abstract

**Objectives:**

To assess the periapical status in patients with osteoporosis (OP) treated with denosumab (D), bisphosphonates (BPs), or not on medication, and to understand if these conditions influence the prevalence and the progression of apical periodontitis (AP).

**Material and Methods:**

Seventy‐six patients with OP alone or treated with D, or BPs, formed the study group (O), and those from 76 patients matched for age and sex, without diseases, and not taking medications, constituted the control (C) in this retrospective case−control study. The data from the complete clinical and radiographic examination, medical history, decayed, missing, and filled teeth (DMFT), and periapical index score (PAI) were recorded for each patient. Wilcoxon rank test, *χ* ^2^, and Student's *t* test were used as appropriate.

**Results:**

The prevalence of AP was similar in O and C. Furthermore, AP was significantly more frequent in root canal‐treated teeth in O patients (*p* = .03).

**Conclusions:**

OP does not appear to be associated with the development of AP. Moreover, the increased prevalence of AP in root canal‐treated teeth in O patients highlights a possible relationship between the healing dynamics of the disease post‐therapy and the patients' medication. A larger sample is needed to confirm these findings.

## 1 INTRODUCTION

Osteoporosis (OP) is a prevalent skeletal disease that affects more than 200 million people worldwide. One in three women over the age of 50 years and one in five men are believed to experience osteoporotic fractures according to the International Osteoporosis Foundation (IOF) (Sözen et al., 2017). Depending on the factors that condition bone metabolism, OP is classified as primary OP, which includes postmenopausal (Type I) and senile OP (Type II), and secondary OP influenced by pre‐existing diseases, medications, and lifestyle (Sözen et al., 2017). Postmenopausal women are the most affected with a growing risk as age increases (Bonnick et al., 2010) because the lack of estrogens and the simultaneous rise in serum levels of the pituitary follicle‐stimulating hormone lead to a stronger osteoclast‐mediated bone resorption (Qian et al., 2016; Trémollieres, 2019). OP is characterized by the reduction of bone mass and the disruption of bone architecture, resulting in an increased risk of fracture (Sözen et al., 2017). The interaction between the receptor activator of nuclear factor kappa B (RANK) and its ligand (RANKL), a member of the tumor necrosis factor (TNF) family, regulates the differentiation and activity of osteoclasts, while osteoprotegerin (OPG) a soluble receptor for RANKL prevents excessive bone resorption. An imbalance of the RANKL/RANK/OPG system is typically present in OP (Braz‐Silva et al., 2019). Bisphosphonates (BPs) and denosumab (D) are antiresorptive and immunomodulatory medications, which represent the current treatment of choice for severe cases of OP (Ruggiero et al., 2014). Despite the different mechanisms of action, they both target the osteoclast and inhibit bone resorption. BPs are pyrophosphate analogs, but with a carbon atom that can have up to two additional side chains, R1 and R2, instead of the oxygen atom. They bind to bone hydroxyapatite and enter osteoclasts, with their consequent inactivation or increased apoptosis, inhibiting the intracellular mevalonate pathway. D is a high‐affinity and highly specific monoclonal antibody for RANKL, capable of stopping the link with its receptor RANK on the surface of osteoclasts and their precursors, preventing their function (Anastasilakis et al., 2018).

In the past 17 years, an increasing number of studies have focused on the most serious adverse effect of these drugs, the medication‐related osteonecrosis of the jaws (MRONJ) (Marx, 2003; Ruggiero et al., 2014). Patients are diagnosed with MRONJ if all the three following characteristics are present: 1. Current or previous treatment with antiresorptive medications or antiangiogenic agents, 2. Exposed bone or bone that can be probed through an intraoral or extraoral sinus tract in the maxillofacial region that has persisted for longer than 8 weeks, 3. No history of radiation therapy to the jaws or obvious metastatic disease to the jaws. The classification of MRONJ consists of four stages: in Stage 0 patients have nonspecific clinical findings, radiographic changes, and symptoms, but no clinical evidence of exposed/necrotic bone which on the other hand is present in Stages 1−3 (Ruggiero et al., 2014; Song, 2019). The major risk for osteonecrosis seems to be related to oncologic patients under high intravenous doses of BPs or subcutaneous D, whereas in osteoporotic patients the incidence is from 0.001% to 0.01% (Song, 2019). Surgical dental treatments and preexistent local infection have been identified as trigger factors. Therefore, dentists need to assess carefully the medical history of the patients considering when the treatment with antiresorptive medications is scheduled or has already started in order to provide proper dental care and prevent MRONJ (Song, 2019).

Apical periodontitis (AP) is an inflammatory condition of the periapical tissues caused by the infection of the root canal system, often expressed by the development of a radiographically visible lesion in the periapical bone (Nair, 2004). The relationship between OP and marginal periodontitis has previously been investigated, with OP patients exhibiting an increased risk of periodontitis as well as an accelerated progression rate of periodontal tissue destruction (Juluri et al., 2015). Furthermore, it has been suggested that the radiographic analysis of the periodontal bone may be considered a screening tool for early signs of OP (Ayed et al., 2018). Studies in the endodontic field have shown that a higher expression of RANKL is related to the severity of AP (Belibasakis et al., 2013; Braz‐Silva et al., 2019; Estrela et al., 2016), and that the cytokines released during periapical inflammation are responsible for more extensive periapical lesions, that become more evident in osteoporotic patients with decreased bone density (Khalighinejad et al., 2016; Lerner, 2006; López‐López et al., 2015). BPs primarily function by blocking the activation of osteoclasts, thereby limiting bone resorption (Anastasilakis et al., 2018); consequently, it may be expected that the osteolytic process, associated with periapical lesions in the osteoporotic patients on BPs, is diminished. When AP was studied in ovariectomized rats, the hypoestrogenic condition of the animals resulted in larger AP lesions (Silva et al., 2020; Wayama et al., 2015; Xiong et al., 2007), and, if on one hand, the administration of BPs limited bone destruction (Rao et al., 2017; Silva et al., 2020; Song et al., 2016; Wayama et al., 2015; Xiong et al., 2007), on the other hand, these medications increased the risk of MRONJ in the presence of pre‐existing AP (Rao et al., 2017; Song et al., 2016; Wayama et al., 2015). AP in osteoporotic patients taking long‐term oral BPs has been shown to heal at a regular rate, after conventional root canal treatment, thus offering an excellent alternative to tooth extraction, a condition significantly more likely to cause MRONJ (Hsiao et al., 2009). Nevertheless, BPs may weaken the immune system (Sabatino et al., 2014), as cases of persistent AP were shown to be more frequent in compromised patients in treatment with BPs for more than 1 year (Dereci et al., 2016). More than 190 million prescriptions for BPs were released globally and the use of D has been recently on the rise (Song, 2019). D is a human monoclonal antibody biologic medication (BM) (Cotti et al., 2014), which blocks the RANKL‐RANK signaling, thus affecting the formation, activity, and survival of osteoclasts. This antiresorptive agent may both limit osteolysis in AP, and affect the healing of periapical lesions due to its immune‐suppressing action (Miyazaki et al., 2014). With the exception of studies focused on MRONJ, possible links between D and endodontic infections have not been investigated to date (Aljohani et al., 2018; Nicolatou‐Galitis et al., 2019; Ruggiero et al., 2014). Notably, in a murine model of experimentally induced periodontitis, anti‐mouse RANKL antibodies proved more effective in preventing bone resorption than a BP (zoledronate) (Kuritani et al., 2018). The aim of this retrospective, case−control study was to assess the periapical status in patients affected by primary OP, either being treated by BPs oral, intravenous (IV), and intramuscular (IM) or D, or not on medication and to observe whether OP as well as BPs or D are associated with the presentation and progression of AP. Medication type, length of treatment, and route of administration of the drugs were also considered.

## 2 MATERIAL AND METHODS

This study was conducted on 76 patients, originally diagnosed with OP and referred to the University Dental Clinic for screening from the Departments of Rheumatology and Orthopedics at the University Hospital, from February 2015 to October 2020, for the assessment of the presence of dental infections. Seventy‐six matched healthy patients, undergoing dental evaluations in the same timeframe, were selected to constitute controls. This protocol was reviewed by the Institutional Ethical Committee of the University Hospital (AOUCA), and it is in compliance with the Helsinki Declaration of 1964, and its later amendments or comparable ethical standards.

### 2.1 Selection of cases

Inclusion criteria for the study group were: male and female; age range 40−80 years; affected by primary OP; in treatment with D, BPs (Oral, IV, and IM), or not in treatment for OP (NM); absence of diabetes, cardiovascular diseases, osteoarticular pathologies different from OP, autoimmune diseases, cancer, and who agreed to participate to the study. Dual‐energy X‐ray absorptiometry (DXA) was used to assess bone mineral density (BMD) and diagnose OP. Patients who were not in the selected age group, or who were under D or BPs, because of different conditions, those taking other medications, or having a history of long‐term use of steroids, chemotherapy, or radiotherapy, with incomplete clinical documentation, or who did not allow their data to be used, were excluded. The control group (C), consisted of individuals who agreed to participate in the study, had no history of OP or any other systemic diseases and were under no medications. They were randomly recruited among patients attending the dental clinic and matched as closely as possible for age, gender and socioeconomic status, and smoking habits with the O patients.

### 2.2 Clinical data collection

Written informed consent for the use of the medical and dental charts was obtained from all patients. All medical records reporting the demographic data, medical history, and medications taken were examined. Detailed information on OP comprised: (a) the date of the first diagnosis of the disease; (b) the medications taken since the diagnosis was established; (c) the duration of treatment with each medication; (d) the medication/s the patient was taking at the time of the dental assessment. The parameters obtained from the dental screening were: (a) number of teeth; (b) presence of soft tissue lesions; (c) caries; (d) conservative and prosthetic restorations; (e) root canal treatments; (f) AP; (g) periodontitis. The routine radiographic investigation for each patient comprised one panoramic and selective periapical radiograph taken in the upper anterior teeth, and in all teeth that presented conservative or prosthetic restorations, AP, or endodontic treatment. The intraoral radiographs were performed by using a film holder for paralleling technique, and exposure times and kilovoltage were set as the film manufacturer suggested.

### 2.3 Acquisition of data

Medical history, diagnostic, and treatment information for each patient were transferred to an Excel file, and all the images were obtained and examined. The data available were used to calculate the number of caries and periapical lesions, the prevalence of AP, the decayed, missing, filled teeth (DMFT) index, the periapical index score (PAI), the quality of root canal treatment and coronal restoration in teeth with AP, and the arithmetic mean of the collected data. The results obtained for O patients and C, and among the subjects in the O subgroups were compared, first between the individuals in treatment and those under no medication, then according to the drug taken. The subgroups were as follows: O not in treatment (NM), O and denosumab (D), O and bisphosphonates (BPs), and O + previous bisphosphonates and current denosumab (BPs + D).

The PAI was assessed by four trained and calibrated endodontists on periapical radiographs. Calibration was determined by the observers assigning the score on 50 periapical lesions twice, with a month interval (weighted kappa values for interoperator agreement, *k *= 0.8). For multirooted teeth, the highest scores assigned to the individual roots were used. If the four examiners did not agree, the highest individual scores were chosen. Furthermore, the same examiners analyzed the quality of coronal restoration and endodontic treatment according to the criteria established by Ng et al. (2011). If one of the components did not respect the standards, the overall quality of treatment of the tooth was considered inadequate.

### 2.4 Statistical analysis

Prevalence of AP was evaluated on the number of individuals and on the number of teeth, comparing O and C, and then the O subgroups. Additionally, the prevalence of AP was calculated by differentiating root canal‐treated teeth from nontreated teeth. Multivariate analysis (logistic regression) was performed distinguishing gender, age, medication taken for the disease, length of time the medications were taken, smoking (defined as yes/no), and the number of teeth, to investigate possible confounding roles on the risk of AP. Differences between groups were assessed with *χ* ^2^, Wilcoxon rank test, and Student's *t* test as appropriate. *p* ≤ .05 was considered statistically significant.

## 3 RESULTS

**Figure 1:**
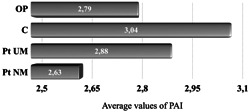
Average values of PAI. PAI, periapical index score.

Osteoporosis group (O) included 76 patients (8 men and 68 women—average age: 64.61 ± 8.09 years) (Table 1). A further distribution in four subgroups comprised: D = 9 patients in treatment with D, BPs = 31 patients with BPs, BPs + D = 11 patients, and NM = 25 patients under no treatment. The average span of time in which the patients were affected by the main disease was 9.48 ± 8.49 years. The average time D was taken was: 2.23 ± 1.58 years, while BPs: 5.29 ± 5.41 years. Patients taking BPs were administered the following medications: Oral BPs: Alendronate; Risedronate; Ibandronate; IM BPs: Clodronate; EV BPs: Neridronate; Zoledronate. The C group was also constituted of 76 individuals (17 men and 59 women—average age 59.67 ± 9.88 years) without OP and major diseases, and not on medications (Table 1). In O 42.1% of patients were affected by AP, whereas the prevalence of AP was 47.4% in C (*p* = .62), and the number of teeth with AP was similar in both groups. No significant differences among the groups were noted with respect to smoking. The DMFT was significantly lower in O (*p* < .01) with O patients presenting with an average of 22.25 teeth each, compared with the average 24.57 teeth of C (*p* = .03) (Table 2). Regarding the prevalence of AP in O patients there were no substantial differences comparing individuals in pharmacological treatment with those under no medications for the disease (*p* = .61). More specifically, there were no significant differences in the prevalence of AP in O subgroups, even if patients on D showed the highest prevalence (66.7%), followed by BP + D (63.6%), NM (36.0%), and BPs (32.3%, *p* = .11). Last, no discrepancies between the groups were observed with respect to the number of teeth affected by AP (Table 3). A higher PAI was detected in C (3.04) rather than in O patients (2.79, *p* = .36) (Figure 1). Multivariate logistic regressions were performed to analyze how gender, age, medications, time the medications were taken, smoking, and the total number of teeth in each individual, set as independent variables, may influence the development of AP: (a) presence of periapical lesion in at least one tooth = 1; (b) absence = 0. Considering all these factors as covariates, individuals in treatment with D resulted at higher risk for AP (OR = 1.83; CI 95% = 1.15–3.37; *p* = .03) (Table 4). All the other variables were not associated with AP. Furthermore, AP was significantly more prevalent in root canal‐treated teeth compared with nontreated teeth in patients with OP, whereas there was no significant difference between treated and nontreated teeth with AP in C (*p* = .03) (Table 5). The quality of root canal treatment and coronal restoration in endodontically treated teeth with AP was judged to be adequate in 56.1% of teeth in O and in 42.9% in C.

**Table 1:** Descriptive data of the sample

	Total	C group	O group
Gender, number (%)			
Overall	152 (100.0)	76 (100.0)	76 (100.0)
Male	25 (16.45)	17 (22.37)	8 (10.53)
Female	127 (83.55)	59 (77.63)	68 (89.47)
Age, mean ± SD	62.14 ± 9.33	59.67 ± 9.88	64.61 ± 8.09
Teeth, number (mean ± SD)	3558 (23.41 ± 5.22)	1867 (24.57 ± 4.12)	1691 (22.25 ± 5.93)
Smoke, number (%)			
Overall	152 (100.0)	76 (100.0)	76 (100.0)
No	122 (80.26)	58 (76.32)	64 (84.21)
Yes	30 (19.74)	18 (23.68)	12 (15.79)

*Note*: Results are presented as percentage frequency (%), mean ± standard deviation.

**Table 2:** Prevalence of AP in O and C groups

	Subjects with AP*	Teeth with AP**	DMFT***	Av. no. of teeth***
Overall				
O	42.1%	0.70 (1.06)	7.75 (4.49)	22.25 (5.93)
C	47.4%	0.67 (0.90)	10.11 (4.08)	24.57 (4.12)
*p* value	.62	.87	**<.01**	.03
Smokers				
O	58.3%	1.17 (1.34)	7.25 (5.03)	22.33 (5.85)
C	61.1%	0.83 (0.99)	9.06 (3.90)	23.06 (4.39)
*p* value	1.00	.47	.27	.97
Non smokers				
O	39.1%	0.61 (0.99)	7.84 (4.42)	22.23 (5.99)
C	43.1%	0.62 (0.88)	10.43 (4.11)	25.03 (3.95)
*p* value	.79	.95	**<.01**	.01

*Note*: Results are presented as percentage frequency, mean (standard deviation).

Abbreviations: AP, apical periodontitis; Av., average number of teeth; DMFT, decayed, missing, and filled teeth.

* *χ* ^2^ test.

** *t* test.

*** Wilcoxon rank test.

**Table 3:** Prevalence of AP in O subgroups

	Subjects with AP*	Teeth with AP**
O		
Patients under medications	45.1%	0.67 (0.89)
Patients under no medications	36.0%	0.76 (1.36)
*p* value	.61	.76
Medications		
D	66.7%	0.78 (0.67)
BPs	32.3%	0.48 (0.81)
BPs + D	63.6%	1.09 (1.14)
NM	36.0%	0.76 (1.36)
*p* value	.11	.41

*Note*: Results are presented as percentage frequency, mean (standard deviation).

Abbreviations: AP, apical periodontitis; BP, bisphosphonate; D, denosumab; NM, not in treatment for OP.

* *χ* ^2^ test.

** *t* test.

**Table 4:** Multivariate logistic regression analysis

	OR	*p* value	95% CI inf. limit	95% CI sup. limit
Gender	1.28	.60	0.51	3.18
Age	0.98	.33	0.94	1.02
D	1.83	.03	1.15	3.37
BPs	0.99	.78	0.88	1.09
Smokers	2.04	.10	0.89	4.85
Av. no. of teeth	1.00	.99	0.93	1.07

Abbreviations: Av., average number of teeth; BP, bisphosphonate; CI, confidence interval; D, denosumab; OR, odds ratio.

**Table 5:** Endodontically treated teeth with AP

	Teeth with RCT and AP
Overall	
O	77.4%
C	54.9%
*p* value	.03
O subgroups	
Patients under medications	73.5%
Patients under no medications	84.2%
*p* value	.58

*Note*: Results are presented as percentage frequency and *χ* ^2^ test.

Abbreviations: AP, apical periodontitis; RCT, root canal treatment.

## 4 DISCUSSION

This retrospective clinical study examined the prevalence of AP in patients with OP and under BPs, D, or no treatment, compared with a C group made of healthy individuals, not on medications. The prevalence of AP in patients with OP was lower than in the controls (42.1% and 47.4%), and the number of teeth affected by AP was similar. Furthermore, no significant differences were reported in the PAI scores between the two groups, while O patients exhibited a lower DMFT (Table 2, Figure 1). Based on these results, primary OP does not appear to worsen the development of AP, regardless of whether the condition is untreated, or treated with therapeutic agents like BPs and D, even though these medications interfere with the turnover of bone and with the host immune system. If we consider OP alone, this observation is not too far from the conclusion drawn in a cross‐sectional clinical study, with a similar sample size, that has described a marginal association between lesions of AP in postmenopausal women with low mineral density conditions (López‐López et al., 2015).

More specifically, Lopez showed that 25% of both osteopenic and osteoporotic women had at least one periapical lesion compared to 7.4% of healthy patients that presented AP (*p* = .06). In the logistic regression adjusted for covariates, BMD correlated marginally significantly with AP (*p* = .05) (López‐López et al., 2015).

The weakness of this association was further confirmed in a review (Khalighinejad et al., 2016). Nonetheless, it is important to note that if the O patients are not receiving any treatment, usually their condition is at an early stage. Another consideration is that the average number of teeth in the O patients of this investigation was lower than in C (22.25 vs. 24.57, *p* = .03), finding that could be related to a lower survival rate in the teeth of osteoporotic patients, as well as to the higher average age of the study group (Table 2). In contrast, Katz and Rotstein in a study based on integrated data of hospital patients with a consequent large sample (42.292) have reported that the prevalence of AP is significantly higher in osteoporotic patients (1.78% vs. 0.52%, OR = 3.36) (Katz & Rotstein, 2021). The strength of these data comes from the large population of the study, but, on the other hand, other possible underlying conditions of the patients were not known, and the diagnostic techniques used to assess AP were not thoroughly specified. Interestingly, we found that AP in the O group was significantly more prevalent in teeth who had received root canal treatment, regardless of whether the patients were under treatment or not, a difference not seen in the control, although the quality of endodontic treatment and restoration, were judged similar (Table 5). This result may imply a change in healing dynamics. It may be hypothesized that in O patients, once the infectious process has started, following RCT, healing becomes more difficult, or that the osteogenic wound healing process is delayed due to altered cellular responses. This may be related to a weaker immune system due to the higher average age of these patients and to the medications used for OP in several cases (Miyazaki et al., 2014). Examining the O subgroups, a higher risk for AP was noticed in the patients taking D; however, these data are based on a small sample and will need to be validated in a larger study (Tables 3 and 4). The prevalence of AP in patients on BPs was the lowest (Table 3), and this is in full agreement with the results from Katz and Rotstein, who went further and identified that specifically Risedronate had the best impact on the smallest incidence of AP (Katz & Rotstein, 2021). It may be speculated that BPs are not relevant for the progression of AP, or alternatively that BPs may contribute to limiting the size of the lesions because of their antiresorptive properties (Rao et al., 2017; Silva et al., 2020; Song et al., 2016; Wayama et al., 2015; Xiong et al., 2007). This observation is also coherent with the results from a retrospective clinical study that reported no difference in the healing of AP between patients under long‐term oral BPs and controls (Hsiao et al., 2009). However, BPs comprise a large range of medications, with the potency of the drug dependent on type, route of administration, frequency, and length of treatment (Dereci et al., 2016). O patients not on medications had only slightly higher values of AP than the individuals under BPs and this finding can be explained by the milder severity of the osteoporotic condition, that did not require treatment (Table 3). Finally, a smoking habit should be considered a weak risk factor for AP, as it was slightly noticeable in the logistic regression analysis, and when the prevalence of AP between smokers and nonsmokers was compared (Tables 2 and 4). Segura‐Egea et al. proposed that smoking may affect the immune system causing a strong inflammatory response, increasing RANKL/OPG ratio with subsequent bone loss, and thus having negative effects on the periapical tissues and the dental pulp (Segura‐Egea et al., 2015). Since the average age of the study group was high, in certain cases, patients with minor comorbidities could not be excluded, and this may represent one of the limitations of the study. Nonetheless, individuals with diabetes, or in treatment with long‐term steroids, chemotherapy, and radiotherapy were not considered. Further, no patients had ever undergone hormone therapies, such as selective estrogen receptor modulators or aromatase inhibitors. The limitation due to the retrospective design of the study makes it difficult to establish a causal relationship between the disease, the medication used, and AP. AP is a multifactorial condition; it is, therefore, complicated to assess all confounding factors that affect this disease. Furthermore, the use of periapical radiographs to evaluate the periapical status of teeth is one of the weaknesses of this study, since a three‐dimensional system such as cone beam computed tomography could provide more precise information on the presence and size of the AP. In addition, the sample size is limited. For this reason, longitudinal studies are required in which variables such as medication, age, and comorbidities can be better controlled. In light of the results obtained, patients with OP should ideally be subjected to an adequate dental screening, before starting medication, while care should be taken to improve the quality and outcome of root canal treatment, which represents the best choice over the extraction of compromised teeth, to minimize the risk of infections and MRONJ (Song, 2019).

## 5 CONCLUSION

Within the limitations of this study, OP does not appear to be associated with the development of AP, even though the increased prevalence of AP in root canal‐treated teeth in O patients highlights a possible relationship between the healing dynamics of the disease post‐therapy and the patients' medication. Treatment with BPs seems to ameliorate the condition of apical health. Due to the preliminary nature of the work, these findings must be interpreted with caution but could be regarded as further mosaic tiles in the field of AP.

## AUTHOR CONTRIBUTIONS

Erika Cadoni and Elisabetta Cotti conceived of the presented idea. Erika Cadoni developed the theory and performed the computations. Francesca Ideo and Erika Cadoni carried out the experiment. Giuseppe Marongiu, Silvia Mezzena, and Luca Frigau developed the theoretical formalism, performed the analytic calculations, and performed the numerical simulations. Francesca Ideo contributed to the interpretation of the results. Elisabetta Cotti and Henry F. Duncan supervised the findings of this study. Erika Cadoni wrote the manuscript with support from Elisabetta Cotti and Henry F. Duncan. Quirico Mela and Antonio Capone helped supervise the project. All authors discussed the results and contributed to the final manuscript.

## CONFLICT OF INTEREST

The authors declare no conflict of interest.

## ETHICS STATEMENT

Approval was obtained from the Institutional Ethical Committee of the University Hospital (AOUCA). The procedures used in this study adhere to the tenets of the 1964 Helsinki Declaration, and its later amendments or comparable ethical standards. Informed consent was obtained from all individual participants included in the study.

## Data Availability

The data that support the findings of this study are available on request from the corresponding author. The data are not publicly available due to privacy or ethical restrictions.
